# Development of a Conventional RT-PCR Assay for Rapid Detection of Porcine Deltacoronavirus with the Same Detection Limit as a SYBR Green-Based Real-Time RT-PCR Assay

**DOI:** 10.1155/2018/5035139

**Published:** 2018-11-06

**Authors:** Lei Ma, Fanwen Zeng, Bihong Huang, Feng Cong, Ren Huang, Jingyun Ma, Pengju Guo

**Affiliations:** ^1^Guangdong Key Laboratory of Laboratory Animals, Guangdong Laboratory Animals, Monitoring Institute, Guangzhou, China; ^2^College of Animal Science, South China Agricultural University, Guangzhou, China

## Abstract

Porcine deltacoronavirus (PDCoV) is a newly discovered coronavirus, which belongs to the family Coronaviridae. It causes watery diarrhea, vomiting, and dehydration in newborn piglets. A sensitive RT-PCR method is urgently required to detect PDCoV infection. In this study, we developed and evaluated a conventional RT-PCR assay and a SYBR green-based real-time RT-PCR assay that targeted the PDCoV *n* gene. Both assays are specific and have the same limit of detection at 2 × 10^1^ copies of RNA molecules per reaction. Eighty-four clinical samples were subjected to both conventional RT-PCR and real-time RT-PCR, and the same positive rate (41.7%) was achieved, which was much higher than the positive rate (26.2%) using a previously described one-step RT-PCR technique. In summary, a conventional RT-PCR technique was successfully established for the detection of PDCoV with the same detection limit as a SYBR green-based real-time RT-PCR assay.

## 1. Introduction

Coronaviruses (CoVs) belong to the family Coronaviridae; they can infect a variety of hosts and cause various illnesses, including respiratory infections and enteric diseases [1–5]. Based on phylogenetic analysis, we can classify CoVs into four major genera: alpha-CoV, beta-CoV, gamma-CoV, and delta-CoV [5, 6]. Porcine deltacoronavirus, which belongs to delta-CoV, was first reported in an epidemiology investigation in Hong Kong [5]. Thereafter, PDCoV was detected and isolated across the world [7–13]. The PDCoV genome is approximately 25-kb long, containing a 5′ untranslated coding region (UTR), replication-associated genes (1a, 1b), a spike (s) gene, an envelope (e) gene, a matrix (m) gene, a nucleocapsid (n) gene, two nonstructural genes (ns6, ns7), and a 3′UTR gene.

Previous studies have reported that *n* gene is highly conserved among all the genes of PDCoV; therefore, the *n* gene has been commonly used as the target for diagnosis [6, 12]. In pigs infected with PDCoV, watery diarrhea was the most common clinical symptom. Some of the newborn piglets infected with PDCoV would succumb to the virus and die eventually [8, 14, 15]. In pigs infected with PDCoV, the symptoms of diarrhea are very similar to that of porcine epidemic diarrhea virus (PEDV) and transmissible gastroenteritis virus (TGEV); therefore, it is difficult to clinically distinguish these viral pathogens.

Various diagnostic methods have been developed to rapidly diagnose PDCoV infection in pigs. Based on detection target, these methods can be divided into virological and serological methods. For the detection of specific antibody, the most commonly used serological assays included virus neutralization (VN) test, indirect fluorescent antibody (IFA) assay, and enzyme linked immunosorbent assay (ELISA) [16–18]. Polymerase chain reaction (PCR) and quantitative PCR (qPCR) are the most commonly used virological methods for the detection of nucleic acid. Various PDCoV-specific RT-PCRs and RT-qPCRs have been developed to detect PDCoV [2, 11, 12]. However, the detection limit of the gel-based RT-PCR for PDCoV detection has not been established [11, 12]. Real-time RT-qPCR was more sensitive than conventional PCR [2, 19]. Although the detection limit of nested PCR was similar to that of real-time RT-PCR, the additional amplification procedure made this test more complicated and easier for contamination.

Although many other methods, such as qPCR and loop-mediated isothermal amplification (LAMP), have been reported [19, 20], conventional RT-PCR is still one of the most commonly used methods for virus detection due to its accessibility and convenience. Thus the aim of the present study was to improve the sensitivity of conventional RT-PCR used for PDCoV detection via screening primers and optimizing PCR reaction conditions. Meanwhile, there is no report about the SYBR green-based RT-qPCR assay for the detection of PDCoV. Thus, a SYBR green-based RT-qPCR assay was developed to compare the detection limit against conventional RT-PCR. Furthermore, conventional RT-PCR and RT-qPCR were used to evaluate clinical samples.

## 2. Material and Methods

### 2.1. Viruses

Porcine deltacoronavirus (PDCoV) strain CHN-GD16-03 and swine acute diarrhea syndrome coronavirus (SADS-CoV) were provided by Professor Jing-Yun Ma, who works at the College of Animal Science, South China Agricultural University, Guangzhou, China. Porcine reproductive respiratory syndrome virus (PRRSV) strain JXA1 was purchased from China Animal Disease Control Center, Beijing, China. Pseudorabies virus (PRV) strain HB-98, classical swine fever virus (CSFV) strain C attenuate vaccine, porcine circovirus type 2 (PCV2) killed vaccine, transmissible gastroenteritis virus of swine (TGEV) attenuate vaccine, porcine epidemic diarrhea virus (PEDV) CV777 attenuate vaccine, porcine rotavirus (RV) NX attenuate vaccine, porcine parvovirus (PPV) CP-99 killed vaccine, and pig foot-and-mouth disease virus (FMDV) killed vaccine were purchased from commercial vaccine companies. Swine influenza virus (SIV) was isolated and preserved in our laboratory.

### 2.2. Generation of RNA Molecular Standard

Out of all the genes in PDCoV, we selected the highly conserved *n* gene for the RNA standard preparation. By using TGuide virus DNA/RNA kit (Tiangen Biotech, Beijing, China), total nucleic acid was extracted from PDCoV Strain CHN-GD16-03. The amplification of specific *n* gene was carried out with PrimeScript™ One-Step RT-PCR Kit (Takara Biotechnology, Dalian, China), which was operated according to product instruction. We designed PCR primers (N-F and N-R) that were used for the amplification of the whole *n* gene (Table 1). The RT-PCR reaction protocol was as follows: reverse transcription step 42°C/30 min, DNA denaturation at 95°C/15 min, 30 cycles of 94°C/30 s, 55°C/30 s, and 72°C/60 s, and additional extension at 72°C for 10 min. The PCR product was purified and ligated into pGEM-T Easy Vector (Promega, Madison, US). One microgram of the plasmid, which was verified by sequencing, was linearized and transcribed* in vitro* by following the manufacturer's instructions provided in RiboMAX™ Large Scale RNA Production Systems (Promega, Madison, US). The transcribed RNA was pretreated with DNase (Promega, Madison, US). Then, it was purified with TRIzol Reagent (Invitrogen, Carlsbad, US). The copy number of the RNA standard was calculated by using routine method.

### 2.3. Primer Design

Oligo 6.0 software (Molecular Biology Insights Inc., CO, US) was used to design primers, which were used in conventional RT-PCR and RT-qPCR assays for the detection of PDCoV. The specificity of primers was determined by NCBI-Blast (https://blast.ncbi.nlm.nih.gov/Blast.cgi). The primers used in this study were included in Table 1.

### 2.4. RT-PCR

After isolating total RNA from samples, it was reverse transcribed into cDNA by following the manufacturer's instructions in PrimeScript™ 1st Strand cDNA Synthesis Kit (Takara Biotechnology, Dalian, China). Using Premix Taq™ kit, the PCR assay was performed under the following conditions: 1 *μ*L each primer (10 *μ*m), 25 *μ*L premix, 1 *μ*L cDNA, and 22 *μ*L distilled water. The reaction conditions were as follows: DNA denaturation at 95°C for 15 min, 30 cycles of 94°C/30 s, 55°C/30 s, and 72°C/60 s; 72°C for 10 min. Using 2% agarose gel, electrophoresis was applied to the resultant PCR product.

### 2.5. RT-qPCR

The synthesized cDNA was used in RT-qPCR assay. Moreover, SYBR Green™ Premix Ex Taq™ II Kit (Takara Biotechnology, Dalian, China) was used for performing RT-qPCR reaction. The final volume of the reaction mixture was 50 *μ*L, and it consisted of the following ingredients: 1 *μ*L each primer (10 *μ*m), 25 *μ*L SYBR Green Premix, 1 *μ*L ROX Reference Dye II, 1 *μ*L cDNA, and 21 *μ*L distilled water. The amplification was performed with an Applied Biosystems 7500 Real-Time PCR System (Thermo Fisher Scientific, MA, US) under the following conditions: 95°C* * for 30 s of initial denaturation, which was followed by 40 cycles of 95°C for 5 s and 60°C* * for 34 s. The conditions of the dissociation step were as follows: 95°C* * for 5 s, 60°C* * for 60 s, and 95°C* * for 15 s.

### 2.6. Sensitivity Test

For sensitivity analysis, the RNA molecular standard was tenfold diluted. The diluted RNA standards were tested by conventional RT-PCR and RT-qPCR techniques. Distilled water was served as the control.

### 2.7. Specificity Test

In this experiment, we evaluated the specificity of the following two assays: RT-PCR and RT-qPCR. For this purpose, PDCoV and other swine pathogens, including SADS-CoV, PRRSV, PRV, CSFV, PCV2, TGEV, PEDV, RV, PPV, FMDV, and SIV, were tested individually by RT-PCR and RT-qPCR techniques. The genome of these viruses was extracted by the aforementioned procedure.

### 2.8. Reproducibility

Using the diluted RNA molecular standards, three independent tests were performed on three different days. Thus, the reproducibility of RT-PCR was determined.

Intraassay and interassay coefficients of variation (CV) were calculated by using threshold cycle (C_t_) values of serially diluted RNA molecular standards, which were detected by RT-qPCR in several replicates. Intraassay coefficient of variation (CV) was determined from the results of three replicates per batch. Interassay coefficient of variation (CV) was determined by testing RNA standards in duplicate on three different days.

### 2.9. Detection of Clinical Samples

We tested 84 clinical samples, including 24 fecal swab specimens, 30 fecal specimens, and 30 intestine specimens; these specimens were collected from a commercial swine farm in Guangdong province, China. All these specimens were tested by both RT-PCR and RT-qPCR techniques in order to evaluate the detection capacity of developed assays. A previously described RT-PCR method was also used to detect the samples [11]. This method was designated as one-step RT-PCR in order to distinguish it from conventional RT-PCR developed in present study.

## 3. Results

### 3.1. Sensitivity of RT-PCR and RT-qPCR

**Figure 1:**
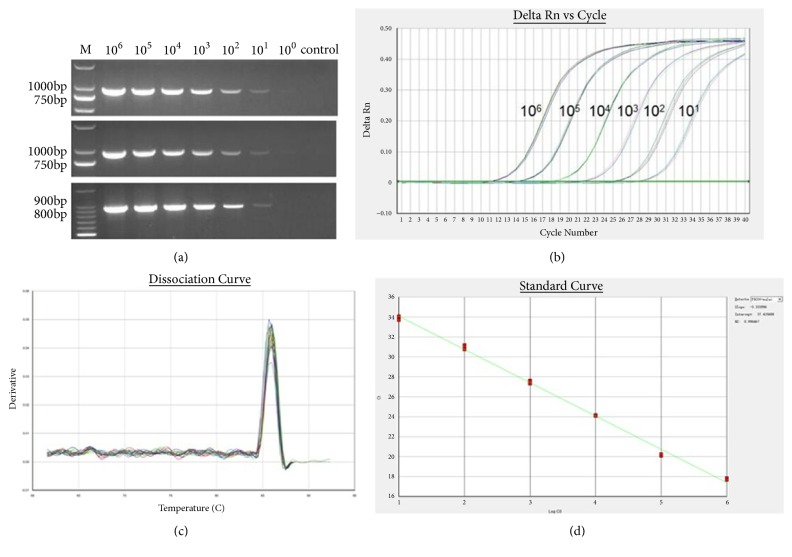
Sensitivity of RT-PCR and RT-qPCR. (a) Amplification of 10-fold dilutions of standard RNA, which was achieved by conventional RT-PCR technique in three different days. (b) Amplification of 10-fold dilutions of RNA standard by RT-qPCR in triplicates. (c) Analysis of dissolution curve of real-time RT-PCR products. (d) A standard curve of RT-qPCR, which was generated by plotting mean C_t_ values versus 10-fold dilutions of RNA standard in triplicates.

To determine the sensitivity of RT-PCR and RT-qPCR assay for PDCoV detection, we used tenfold dilutions that ranged from 2 ×10^6^ to 2 ×10^0^ copies/*μ*L of RNA molecular standard. As shown in Figure 1(a), the limit of detection of RT-PCR was 2 × 10^1^ copies; no amplification product was produced by testing 2 × 10^0^ copies of RNA and distilled water. The results of other primers were not presented, which showed low sensitivity.

Using RT-qPCR technique, we could detect as few as 2 × 10^1^ copies of molecular RNA (Figure 1(b)). Melting dissociation analysis on the qPCR products showed the same melting temperature (T_m_) which was 86.0°C (Figure 1(c)). With an R^2^ value of 0.996, we obtained a standard curve by plotting threshold cycle (C_t_) versus RNA copy numbers. This indicates that the amplification efficiency of RT-qPCR technique was indeed remarkable (Figure 1(d)).

### 3.2. Specificity of RT-PCR and RT-qPCR

**Figure 2:**
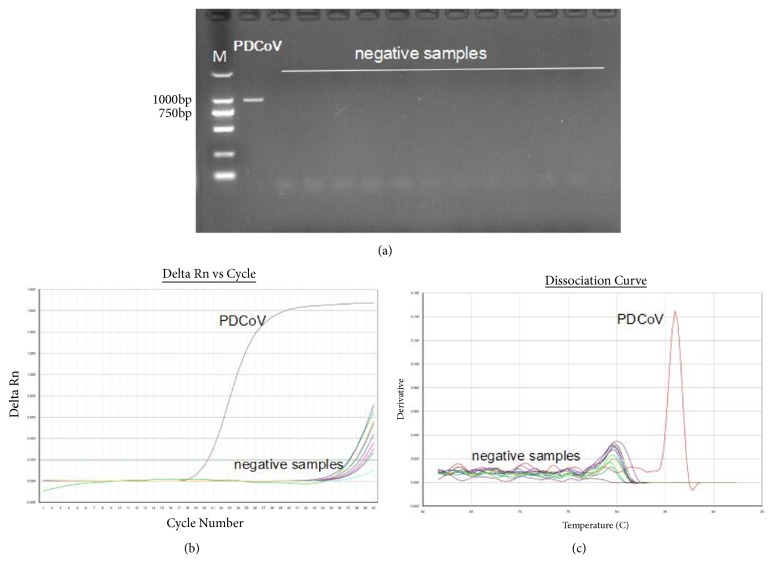
Specificity of RT-PCR and real-time RT-PCR assay. PDCoV and negative samples were detected by RT-PCR and RT-qPCR. Negative samples included SADS-CoV, PRRSV, PRV, CSFV, PCV2, TGEV, PEDV, RV, PPV, FMDV, SIV, and distilled water. (a) Only PDCoV genome was amplified in RT-PCR. (b) PDCoV was found to have a strong fluorescence signal under RT-qPCR; the C_t_ values of negative samples were greater than 35. (c) Melting peak was observed at 86°C for only PDCoV in RT-qPCR.

We had to determine whether RT-PCR and RT-qPCR assays had the specificity for PDCoV detection; therefore, several viral pathogens of swine (RV, PRV, FMDV, PEDV, PRRSV, CSFV, PCV2, TGEV, SIV, PPRV, and SADS-CoV) were tested by RT-PCR and RT-qPCR assays. Only PDCoV target gene was amplified by RT-PCR and visualized by agarose electrophoresis (Figure 2(a)). PDCoV was found to have a strong fluorescence signal under RT-qPCR; the C_t_ values of negative samples were greater than 35 (Figure 2(b)). Specific melting peaks are shown for only PDCoV detected by real-time RT-PCR (Figure 2(c)). These results indicate that both of these two assays showed good specificity.

### 3.3. Reproducibility

Total three independent tests were performed on three different days to evaluate the reproducibility of RT-PCR; the sensitivity of the three trials was the same (Figure 1(a)).

Intra- and interassay coefficients of variation (CV) of RT-qPCR were calculated by determining the C_t_ values of multiple replicates.  Table 2 shows that both intra- and interassay variability were below 5%. This indicates that real-time RT-PCR had good reproducibility.

### 3.4. Detection of PDCoV in Clinical Samples

In this experiment, a total of 84 clinical samples were simultaneously detected by conventional RT-PCR and real-time RT-PCR. A one-step RT-PCR targeting the *n* gene of PDCoV described in a previous study was also used to detect the samples.  Table 3 presents the positive rates of PDCoV, which were detected by three methods in these samples. Both conventional RT-PCR and real-time RT-PCR techniques showed an equally positive percentage for PDCoV, which was consistent with the sensitivity of the two assays. The PDCoV-status of these samples determined by conventional RT-PCR was 100% in agreement with that of RT-qPCR. However, when these samples were tested by one-step RT-PCR, the overall positive rate was only 26.2% for PDCoV. This indicates that one-step RT-PCR was significantly less sensitive than the other two assays.

## 4. Discussion

It is important to note that PDCoV is a newly discovered coronavirus, which is circulating across the world [2, 9, 13, 14, 21]. Low feed remuneration is observed in pigs infected with PDCoV pathogens; the impact of PDCoV was more severe in infected newborn piglets, which ultimately succumbed to the virus and died. Currently, there are no effective treatments and vaccines against PDCoV infection. To monitor the health of pig herds, scientists need to develop a rapid diagnosis method that is suitable for most laboratories in China. A rapid and accurate diagnosis would be helpful to identify and segregate infected animals in a timely manner. Therefore, the main objective of present study is to establish the conventional RT-PCR and SYBR green-based RT-qPCR methods with high sensitivity for PDCoV detection. The sensitivity of developed conventional RT-PCR is same as that of RT-qPCR. After comparing the results of clinical samples analyzed by both assays, we concluded that these methods were sensitive, rapid, reliable, and cost-effective in nature.

Several kinds of novel amplification methods, such as loop-mediated isothermal amplification (LAMP), insulated isothermal PCR (iiPCR), and probe-based real-time PCR, have been developed to detect PDCoV with better sensitivity [19, 20]. Nevertheless, conventional RT-PCR is still widely used for detecting PDCoV [11, 12]. Although previous studies have used conventional RT-PCR to detect PDCoV, the detection limits of these assays have not been defined till date [11, 12]. A previous report has described that the detection limit of LAMP method was 10 copies, which was 100 times more sensitive than conventional RT-PCR [20]. This indicates that RT-PCR can detect PDCoV with a detection limit of 1000 copies. Recently, nano-particle-assisted PCR assays were used to increase the sensitivity of conventional PCR [22–24]. In the detection of duck Tembusu virus, the detection limit of nano-particle-assisted PCR was 1.8 × 10^2^ copies [23]. This indicates that the sensitivity of nano-particle-assisted PCR was 10-fold more than that of conventional PCR assay [23]. In theory, PCR assay can detect as few as 1 DNA/RNA molecule. However, the limit of detection for conventional PCR commonly could not achieve theoretical value because of various factors, such as primer design and reaction inhibitor in genome extract.

In present study, our main objective was to ensure that conventional RT-PCR could detect PDCoV with better sensitivity. The conservation of the target gene is important to the broad reactivity of the RT-PCR assay for detection of heterologous strains. The PDCoV *n* gene was demonstrated to be highly conserved and chosen to be the target gene of RT-PCR and RT-qPCR in many studies [6, 12]. Thus, the *n* gene was selected as the target gene in our study. To improve the sensitivity of the conventional RT-PCR, we adjusted several primer pairs and optimized reaction conditions. It was found in our study that the sensitivity of the conventional RT-PCR assay could be significantly improved with selected primers. However, modulation of reaction conditions, such as annealing temperature and primer concentrations, had no effect on the performance of the assay. The optimal sensitivity of RT-PCR was 2 × 10^1^ copies by using the primers PCR-F/R for PDCoV detection, which was equal to that of SYBR green-based RT-qPCR. The result was confirmed by the tests carried out in three independent days. The conventional RT-PCR had several advantages against the RT-qPCR as follows: RT-PCR does not require an expensive device for operation; moreover, RT-PCR is a cost-effective technique. In present study, it was proved that the sensitivity of RT-PCR was significantly improved in resource-limiting settings. Therefore, the high sensitivity of this method will reduce false negative results. The sequences of PDCoV *n* gene have been used to generate the phylogenetic tree to analyze the epidemiology of PDCoV [13]. In our study, the *n* gene products were approximately 950 nt in length; these gene products could be sequenced directly, providing a robust tool for molecular epidemiology study.

Furthermore, 24 fecal swab specimens, 30 feces specimens, and 30 intestine specimens were simultaneously tested by conventional RT-PCR and SYBR green-based RT-PCR. The detection result of the conventional RT-PCR assay was 100% in agreement with that of the RT-qPCR assay, indicating the high sensitivity of the conventional RT-PCR assay. The positive rates detected by conventional RT-PCR and RT-qPCR were both 41.7%. However, when the samples were tested by one-step RT-PCR [11], an assay reported in a previous study, the positive rate was only 26.2%. These results demonstrated that the previously reported conventional RT-PCR with low sensitivity would underestimate the prevalence of PDCoV infection in pig herd. Considering that most testing laboratories are equipped with thermocyclers rather than sophisticated quantitative PCR instruments, the highly sensitive conventional RT-PCR is more suitable for extensive applications in laboratories, which are specifically designed for PDCoV detection and surveillance.

## 5. Conclusion 

In this study, a highly specific and sensitive conventional RT-PCR assay was successfully established for the detection of PDCoV, with the same detection limit of 2 × 10^1^ copies molecule as a SYBR green-based real-time RT-PCR assay. The performance of the conventional RT-PCR assay was validated using 84 clinical samples. The improved RT-PCR provided a cost-effective and highly sensitive method for the diagnosis of PDCoV infection, which is epidemic in pig herds across the world.

## Figures and Tables

**Table 1:** Primers used in this study.

Name	Sequence
N-F	ACGCTGCTGATTCCTGCT
N-R	GCTACTCATCCTCAGTTTCGTG
PCR-F	TGGAACTGACCCGGATGTTG
PCR-R	GCGTACACCCTAGTGGCTTC
qPCR-F	TTCCTATGGAGATGACCTATTAATTGGAAC
qPCR-R	TCAGAGGAAAGGTGGTGGTCTTGTTGGCAG

The primer N-F/R was used for the amplification of whole *n* gene. The primer PCR-F/R was used for performing conventional RT-PCR. The primer qPCR-F/R was used for performing RT-qPCR.

**Table 2:** Intra- and interassay variability.

RNA standard	Intraassay/CT	CV (%)	Interassay/CT	CV (%)
(copies/*μ*L)				
10^6^	14.20±0.09	0.61	14.35±0.12	0.84
10^5^	17.31±0.10	0.58	17.42±0.15	0.86
10^4^	21.41±0.03	0.16	21.64±0.11	0.51
10^3^	24.77±0.21	0.84	25.27±0.26	1.03
10^2^	28.30±0.16	0.55	28.55±0.22	0.77
10^1^	31.74±1.06	3.35	32.34±0.46	1.42

**Table 3:** Detection of PDCoV from clinical samples, which were analyzed by one-step RT-PCR, conventional RT-PCR, and SYBR green-based real-time RT-PCR.

Samples	Number	Number of positive samples		
		One-step	Conventional	Real-time
		RT-PCR	RT-PCR	RT-PCR
Feces	24	7 (29.2 %)	11 (45.8 %)	11 (45.8 %)
Fecal swab	30	9 (30.0 %)	13 (43.3 %)	13 (43.3 %)
Intestine	30	6 (20.0 %)	11 (36.7 %)	11 (36.7 %)
Total	84	22 (26.2 %)	35(41.7 %)	35(41.7 %)

## Data Availability

The data used to support the findings of this study are available from the corresponding author upon request.
